# Poor Man’s 1000 Genome Project: Recent Human Population Expansion Confounds the Detection of Disease Alleles in 7,098 Complete Mitochondrial Genomes

**DOI:** 10.3389/fgene.2013.00013

**Published:** 2013-02-28

**Authors:** Hie Lim Kim, Stephan C. Schuster

**Affiliations:** ^1^Center for Comparative Genomics and Bioinformatics, Pennsylvania State UniversityUniversity Park, PA, USA; ^2^Singapore Centre on Environmental Life Sciences Engineering, Nanyang Technological UniversitySingapore

**Keywords:** mitochondrial disease alleles, human population history, disease allele frequency, human population expansion, mitochondrial variants

## Abstract

Rapid growth of the human population has caused the accumulation of rare genetic variants that may play a role in the origin of genetic diseases. However, it is challenging to identify those rare variants responsible for specific diseases without genetic data from an extraordinarily large population sample. Here we focused on the accumulated data from the human mitochondrial (mt) genome sequences because this data provided 7,098 whole genomes for analysis. In this dataset we identified 6,110 single nucleotide variants (SNVs) and their frequency and determined that the best-fit demographic model for the 7,098 genomes included severe population bottlenecks and exponential expansions of the non-African population. Using this model, we simulated the evolution of mt genomes in order to ascertain the behavior of deleterious mutations. We found that such deleterious mutations barely survived during population expansion. We derived the threshold frequency of a deleterious mutation in separate African, Asian, and European populations and used it to identify pathogenic mutations in our dataset. Although threshold frequency was very low, the proportion of variants showing a lower frequency than that threshold was 82, 83, and 91% of the total variants for the African, Asian, and European populations, respectively. Within these variants, only 18 known pathogenic mutations were detected in the 7,098 genomes. This result showed the difficulty of detecting a pathogenic mutation within an abundance of rare variants in the human population, even with a large number of genomes available for study.

## Introduction

The human population has recently expanded to about seven billion, according to the last census (Roberts, 2011). Studies inferring the demographic history of human populations from genetic data have also shown an increase in effective population size, especially for Europeans and Asians (Gutenkunst et al., 2009; Gravel et al., 2011). Interestingly, the estimated growth rate of effective population size increased as the number of samples analyzed increased (Keinan and Clark, 2012). This finding supports the concept that larger samples can better capture the rare variants in the population, and thus, that sample size could affect the estimation of the demographic history of the human population. Broad sampling is therefore necessary to the accuracy of any study of the history of modern humans.

Rapid population growth also results in an excess of rare variants, which have recently been the focus of many studies of the identification of disease-related variants (Manolio et al., 2009; Cirulli and Goldstein, 2010). Several researchers have detected the association of rare variants with particular diseases (Nejentsev et al., 2009; Calvo et al., 2010; Johansen et al., 2010). For such identification, a large sample size is necessary: the low frequency of rare variants reduces statistical power for detecting a significant association between any single rare variant and a specific disease (Bansal et al., 2010). In addition, the frequency of rare variants can depend upon sampling biases (Keinan and Clark, 2012), since such variants have so recently appeared.

Extensive examination of the distribution of rare variants within subpopulations of humans would greatly contribute toward the development of the analytical strategies needed to identify those rare variants responsible for specific diseases. Relative to this issue, the mitochondrial (mt) genome can be an attractive subject to study. The 16.5 kb sized genome allows us to sequence whole genomes within large samples. The haploid genome of mtDNA allows clear identifications of subpopulations of individual genomes. Further, the human diseases caused by mutations in mtDNA have been well studied. Mutations in mtDNA can play a role in mt dysfunction that leads to energy deficiencies in the cells of our bodies (Wallace, 2010). Several mt diseases have been linked to maternally inherited mutations, for example, Leber’s hereditary optic neuropathy (LHON), mt encephalomyopathy, and Leigh syndrome (Wallace, 2010). Those diseases are characterized by degenerative phenotypes that typically include vision loss, muscle weakness, cardiomyopathy, and dementias.

On the other hand, a high mutation rate of the mtDNA (Shigenaga et al., 1994; Bogenhagen, 1999) means that somatic cells accumulate mutant mtDNA over the life of an individual. The accumulation of these mt mutations could relate to aging and age-related diseases, such as diabetes, obesity, cancers, heart disease, and Alzheimer’s disease (van den Ouweland et al., 1992; Wallace, 2005; Czarnecka and Bartnik, 2011). Because offspring can inherit only mtDNAs passed through germ-line cell proliferation along the maternal lineage (Giles et al., 1980; Bergstrom and Pritchard, 1998), the mutant mtDNA in somatic cells is not transmitted to the next generation.

In this study, we focused on transmitted mutations to ascertain their behavior during recent evolution of the human population; we also investigated effects of the population history of humans on the identification of disease-related mutations among mt variants in the given dataset. We analyzed 7,098 whole mt genomes and determined the demographic model of the human population history of the mt genomes, based on the genetic diversity of the 7,098 mt genomes. Under an assumption of this demographic model, we simulated mt genome sequences and estimated the frequency of deleterious mutations in the population.

## Materials and Methods

### Mitochondrial genome sequences

More than 8,000 complete *Homo sapiens* mt genome sequences were downloaded from GenBank. Genomes that contained gaps longer than 300 bp were subsequently filtered out, most of which had unsequenced control regions. After filtering, we analyzed 7,098 genomes, including the Cambridge reference genome (NC_012920). We have included a list of the accession numbers of these 7,098 genomes in the Supplementary Material.

### Population genetic analyses

The 7,098 genomic sequences were aligned using MAFFT (Katoh et al., 2005). We aligned the two control regions and the non-control intervals independently and then combined them into one alignment. We identified sites having at least two different nucleotides as single nucleotide variants (SNVs). (The indel and heteroplasmic sites were not counted as variants.) We used the software HaploGrep (Kloss-Brandstätter et al., 2011) to identify haplogroups.

### Demographic models

**Figure A1:**
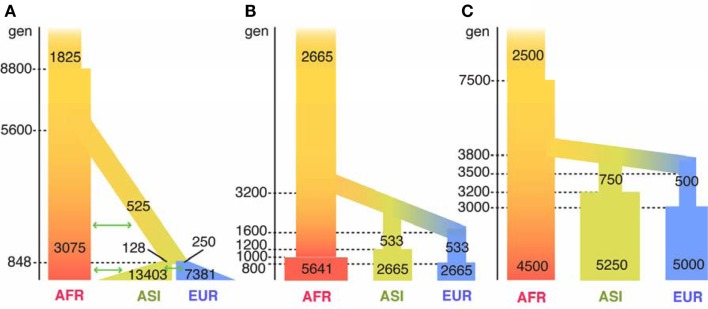
**Illustrations of three human demographic models**. The parameters of the three models were illustrated in the figure. Time flows from past (top) to present (bottom) and shown at the left side of bars. The width of bars represents the size of effective population. The parameters were originally estimated from the nuclear genomes and were modified for mt genomes, under an assumption of that the sex ratio is 1:1. Therefore, the population size is one fourth of that for the nuclear genome. The ms command line for the three models is following. **(A)** Gutenkunst et al.’s (2009) model: ms7098 1000 -t 63.31 -I 3 685 2658 3755 -en 0.1379 3 0.0813 -en 0.1379 2 0.0415 -g 3 33.73 -g 2 24.55 -eg 0.1379 2 0.0 -em 0.1379 2 3 0.5904 -em 0.1379 3 2 0.5904 -em 0.1379 1 2 0.1169 -em 0.1379 2 1 0.1169 -em 0.1379 3 1 0.1845 -em 0.1379 1 3 0.1845 -ej 0.1379 3 2 -en 0.9106 2 0.1707 -em 0.9106 1 2 0.1538 -em 0.9106 2 1 0.1538 -ej 0.9106 2 1 -en 1.4309 1 0.5935. **(B)** Voight et al.’s (2005) model: ms7098 1000 -t 63.31 -I 3 685 2658 3755 -en 0.07 3 0.47 -en 0.09 1 1 -en 0.11 2 0.47 -ej 0.14 3 2 -en 0.14 3 0.09 -ej 0.28 2 1 -en 0.28 2 0.09. **(C)** Marth et al.’s (2004) model: ms7098 1000 -t 63.31 -I 3 685 2658 3755 -en 0.33 3 1.11 -en 0.36 3 1.39 -en 0.39 3 0.11 -ej 0.39 3 2 -en 0.42 2 0.17 -ej 0.42 2 1 -en 0.83 1 1.

In order to determine a suitable demographic model for the human mt population, we tested three human demography models (Marth et al., 2004; Voight et al., 2005; Gutenkunst et al., 2009), using the ms coalescent simulation program (Hudson, 2002). We tuned the simulation parameters based on the assumption that the effective population size of the human mt genome was a quarter of that of the human nuclear genome (Hartl and Clark, 2007). (See Figure A1 in Appendix for the parameters and the ms command lines). For each model, we simulated 1,000 sets of 7,098 genomes. The sample size of each population was identical to the number of genomes in each haplogroup.

### Forward simulations

To ascertain the frequency of a deleterious mutation within a population during demographic events, we needed to trace such mutations by forward simulations. Forward simulations generated mt genome sequences with the same length as the human mt reference genome (16,569 bp), based on the Gutenkunst et al. (2009) model. We excluded migration events between populations in the model because the expected migration rate was low, and the lack of recombination in mtDNA meant the effect of such migration was likely very small.

The African demographic model contained only one population expansion (*N_e_* from 1,825 to 3,075) 8,800 generations ago, and no demographic event up to that time point. The time of 8,800 generations is longer than the fixation time (2*N_e_* = 6,150 generations) of a neutral mutation in a population. Because a deleterious mutation is eliminated within a population in less time than a neutral mutation, 8,800 generations is long enough to trace the frequency of any deleterious mutation. Therefore, we simulated an ancestral population of *N_e_* = 3,075, and ignored history prior to 8,800 generations. To save time in reaching the state of population equilibrium, we used the FREGENE program (Chadeau-Hyam et al., 2008) to generate the ancestral population.

Basically we repeated a process of mutation, random sampling, and selection in each generation. In each generation, mutations occurred at a rate of μ = 2.0e−07 per generation per site without reverse mutation in the ancestral and AFR population. The simulation was tuned to generate the expected diversity (the observed nucleotide diversity of African).

According to the assumed demographic model, African and non-African populations split 5,600 generations ago. For the ancestral population of ASI and EUR, we randomly selected 525 genomes from the ancestral population (*N_e_* = 3,075). During 4,752 generations, non-African evolved with the mutation rate of μ = 7.0e−07 per generation per site, without reverse mutation, which was adjusted for the extent of expected diversity. The mutation rate was set lower than the African rate, which was adjusted for the extent of diversity expected for non-African. Note that the *rate* of mutation does not affect the *frequency* of a mutation. A defined mutation rate was needed only for the process of generating new mutations.

At the point in time of 848 generations ago, 128 ASI and 250 EUR genomes were randomly selected from the ancestral population of non-African (*N_e_* = 525). Since the two populations evolved independently up to the present, from the 848 generations ago, practically, our simulations were separately performed for each of three populations.

A frequency of a mutation was traced while negative selection operated on the mutation during our random sampling. Such a deleterious mutation was randomly selected among new mutations for each population, and its frequency was traced until it was eliminated or fixed in the population. The simulation then randomly selected a new mutation in the next generation. The selection coefficient for negative selection was supposed to be consistent throughout the evolution of the population. Each simulation tested for each of the various selection coefficients ranging from neutral evolution to strong negative selection, 0 ∼ 0.1. For the sake of a clear modeling of negative selection, we assumed that only a single deleterious mutation existed at any one time. The most important parameter for determining the frequency of a deleterious mutation was the population growth rate. The AFR effective population size has not changed, while the population size in the ASI and EUR populations increased at a rate of 0.55 and 0.4% per generation, respectively. We recorded the frequency within each of three populations at the last or present generation if at least one genome carried the mutation. For each selection coefficient, we collected 10,000 frequency data of the mutations in each of three populations.

## Results

### The single nucleotide variants in 7,098 human mitochondrial genomes

We analyzed 7,098 complete mt genomes after filtering non-complete genomes among the retrieved sequences from GenBank (see Materials and Methods). Since our aim was to study the inherited variants, we intended to exclude any somatic mutations contained in the retrieved sequences. Also, as many of the mt genomes were generated via the sequencing of PCR products, it is likely that some mt sequences in the GenBank may contain the result of technical errors (Yao et al., 2009). It was therefore challenging to distinguish rare variants from sequencing artifacts. To do so, among all variants found in the 7,098 genomes, we used only SNVs, with the exception of indel and heteroplasmic sites. The SNVs were then grouped into two categories: the first (Dataset 1) consisted of all SNVs identified from the 7,098 genomes, and the second (Dataset 2) contained all SNVs with the exception of singletons which appeared only once among the samples (Table 1). While Dataset 1 is likely to include more errors, Dataset 2 might have lost many rare variants as a consequence of our attempts at error reduction.

**Table 1:** **The number of SNVs of the 7,098 human mt genomes for each region**.

		Dataset 1		Dataset 2	
		Number	Proportion (%)	Number	Proportion (%)
**Position**					
Total		5,554		3,895	
Multi-allelic		517	9	183	5
**SNVs**					
Total		6,110		4,092	
Control region		663	11	484	12
Non-coding		27	0	20	0
RNA genes		882	14	528	13
Protein-coding	Non^a^	1,515	25	882	22
	Syn^b^	3,023	49	2,188	53

*^a^Non-synonymous SNVs*.

*^b^Synonymous SNVs*.

In total, 5,554 nucleotide positions were identified as having nucleotide variations. Among the positions, we detected 517 multi-allelic positions that had at least three variations. Those multi-allelic positions were considered as multiple variants, for example, a tri-allelic position was consistent with two SNVs. We counted 1,073 SNVs for the 517 positions; 6,110 SNVs were finally identified in 5,554 nucleotide positions. These 6,110 SNVs were consistent with Dataset 1. Dataset 2 contained 4,092 SNVs found in 3,895 nucleotide positions. Among those positions, 183 had at least three variations and 380 SNVs were identified (Table 1). Of the Dataset 1 SNVs, 663 (11%) and 5,447 (89%) were located in the control and remaining regions respectively. Similar ratios were found for Dataset 2: 12 and 88% for the corresponding regions respectively (Table 2).

**Table 2:** **The genetic diversity of the 7,098 human mitochondrial genomes**.

	Region	Length (bp)	*S*^a^	θ^b^ (%)	Π^c^	π^d^ (%)	Max^e^
Dataset 1	All	16,569	6,110	3.90	39.8	0.24	123
	Control	1,122	663	6.26	9.9	0.88	32
	Remain	15,447	5,447	3.73	29.9	0.19	98
Dataset 2	All	16,569	4,092	2.61	39.3	0.24	122
	Control	1,122	484	4.57	9.8	0.88	32
	Remain	15,447	3,608	2.47	29.5	0.18	96

*^a^The number of segregating sites (equal to the number of SNVs)*.

*^b^The number of segregating sites per site, Watterson’s θ (Waterson, 1975)*.

*^c^The average number of pairwise nucleotide differences, Nucleotide diversity per genome (Nei and Li, 1979)*.

*^d^The nucleotide diversity per site*.

*^e^The maximum number of pairwise nucleotide differences*.

**Figure 1:**
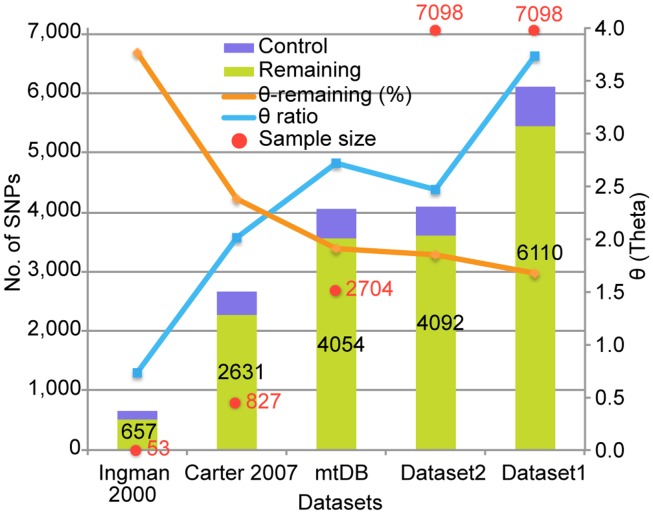
**Comparison of the number of identified SNVs to previous studies**. The *X*-axis indicates five datasets from Ingman et al. (2000), Carter (2007), mtDB (http://www.mtdb.igp.uu.se/), and our dataset 1 and 2. A red dot with a number shows the number of genomes used in the studies or database. The bar graph represents the number of SNVs identified in each dataset, and the number in the bar is the SNV number. The orange lines indicate the number of segregating sites per site (θ) for the remaining region in mtDNA. The blue lines indicate the ratio of the θ value for the control versus the remaining regions.

The SNVs identified in this study are the largest datasets of the human mt variants studied so far (Figure 1). As the sample size increased, the number of identified SNVs also increased. The similarity of the number of SNVs in mtDB^1^ to the number in Dataset 2 supports the existence of those SNVs in the human population. In addition, the large difference in the numbers of SNVs between Dataset 1 and 2 can be explained by the existence of singleton SNVs; that is, those which appeared only once in the 7,098 genomes. Although the number of SNVs was corrected by the sample size to Waterson’s θ (Waterson, 1975), the θ value for the entire region still increased, except in Dataset 2. On the other hand, the ratio of the θ value for the control versus the remaining regions decreased as the sample size increased. This suggests that the number of SNVs increased in the remaining region rather than the control region as the sample size increased. Therefore, the large number of Dataset 1 SNVs resulted from a large number of singletons and increased variant capture in the remaining region due to the large sample size.

### Population structures of 7,098 human mitochondrial genomes

The genetic diversity of the 7,098 human mt genomes could be represented by the nucleotide diversity (Π: per genome, π: per site; Nei and Li, 1979), which represents the nucleotide difference between two randomly chosen genomes. Nucleotide diversity is less likely to be affected by rare and/or erroneous SNVs, because it considers allele frequency in a population. The Π value of the 7,098 genomes was 39.8 and 39.3 for Dataset 1 and 2, respectively, and the π value was 0.24% for both datasets (Table 2). However, overall nucleotide diversity can be biased by population samplings. In order to examine the distribution of origin of the samples, we identified haplogroups for the 7,098 genomes. Every known haplotype was found in the genomes, indicating that the assembled mt database of 7,098 mt genomes represents sufficiently deep sampling of the human population worldwide. Because our SNV dataset included many novel and/or rare SNVs, most of the genomes were unique and newly identified haplotypes. For the 7,098 genomes in this study, we determined only macro-haplogroups identifying three major populations of African, Asian, and European humans. Our simplifying assumption was that the three representative haplogroups (assigned L, M, and N) correspond to the African, Asian, and European origin populations respectively (Wallace et al., 1999). The 7,098 genomes consisted of 685 (10%) of the L haplogroups (African, AFR), 2,658 (37%) of the M haplogroups (Asian, ASI), and 3,755 (53%) of the N haplogroups (European, EUR; Table A1 in Appendix). AFR is most underrepresented in our dataset.

**Figure 2:**
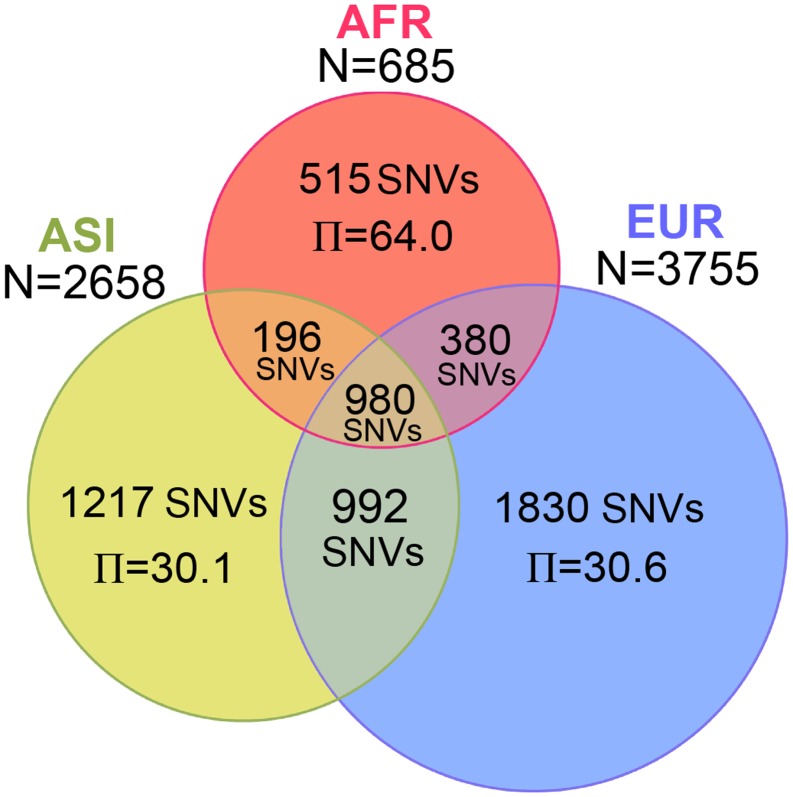
**Number of SNVs and nucleotide diversity for three populations**. The Venn diagram represents the number of SNVs identified in AFR, ASI, and EUR. Inside the diagram, the number of SNVs and the nucleotide diversity per genome (Π) are shown in the corresponding area.

Within AFR, ASI, and EUR, the numbers of SNVs were 2,071, 3,385, and 4,182 respectively, for Dataset 1 (Figure 2). The number of SNVs apparently depended upon the sample size. Therefore, it is not surprising that EUR, having the largest sample size, had the largest number of SNVs. The Π value should not be affected by the sample size; therefore we can compare diversity among the three populations. The Π value within AFR, having the smallest sample size and number of SNVs, showed the largest nucleotide diversity (64.0). The nucleotide diversity values within ASI (30.1) and within EUR (30.6) were both less than half the AFR value (Table A2 in Appendix). The Π value between AFR and ASI (57.9) was similar to that of the diversity between AFR and EUR (60.3), and both these values were even marginally smaller than the Π value within AFR itself. The overall distribution of Π values for Dataset 2 was similar (Table A2 in Appendix).

**Figure 3:**
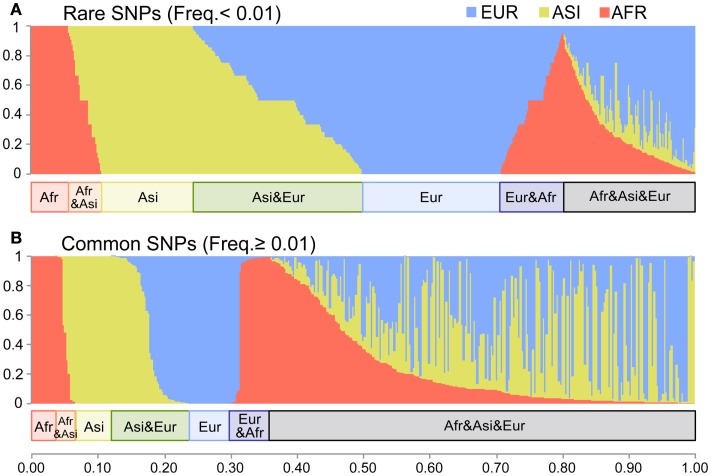
**The distribution of the shared variants**. The *X*-axis indicates each SNV of Dataset 2. The *Y*-axis indicates the fraction of the copies of the non-reference allele present in each of the three populations. The length of red, yellow, and blue in each bar shows the fraction of AFR, ASI, and EUR respectively. **(A)** The rare SNVs with frequency ≤ 0.01 (3,709 SNVs). **(B)** The common SNVs with frequency > 0.01 (383 SNVs).

**Figure A2:**
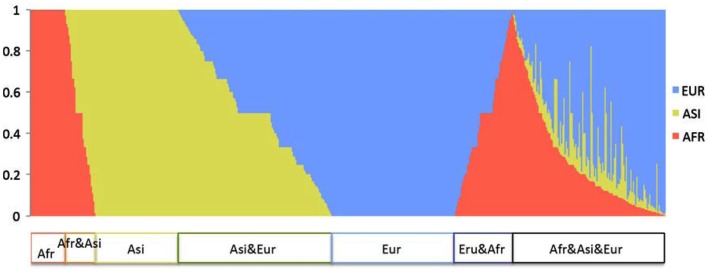
**Sharing distribution of SNVs across populations**. The *X*-axis indicates each of all Dataset 2 SNVs, 4,092 SNVs, and the *Y*-axis indicates the proportion of three populations in the genomes carrying a non-reference allele for each SNV. Red, yellow, and blue bars show the proportion of AFR, ASI, and EUR, respectively.

To show the divergence among populations, we examined their shared variants. We used Dataset 2 for this analysis because all singleton SNVs were population-specific. Among the Dataset 2 SNVs, 62% shared in at least two populations (Figure A2 in Appendix). In the AFR, ASI, and EUR SNVs, the proportions of the population-specific variants were 12, 20, and 25% respectively. We categorized Dataset 2 SNVs into the common (frequency > 0.01) and the rare (frequency ≤ 0.01) groups in order to compare the sharing of variants between the two. Most SNVs (3,709; 91%) fell into the rare SNV group (Figure 3A); only 383 SNVs (9%) were in the common group (Figure 3B). The proportion of population-specific variants was higher in the rare group (40%) than in the common group (15%). The proportion of shared variants across populations was very low among the rare SNVs. In contrast, most common SNVs (85%) shared at least two populations.

It is likely that the population-specific variants were generated very recently after population splits, while the shared common variants have existed for a long time, preceding the population splits, and their frequency increased across populations. The lack of shared variants between populations suggests divergent subpopulations within the human population. On the other hand, the large proportion of rare variants and the small amount of diversity in non-AFR is likely to be a result of recent non-AFR population expansions, as is known to be the case for nuclear variants (Gravel et al., 2011). The demographic history of the human population can play an important role in the distribution of variants in mt genomes.

**Figure 4:**
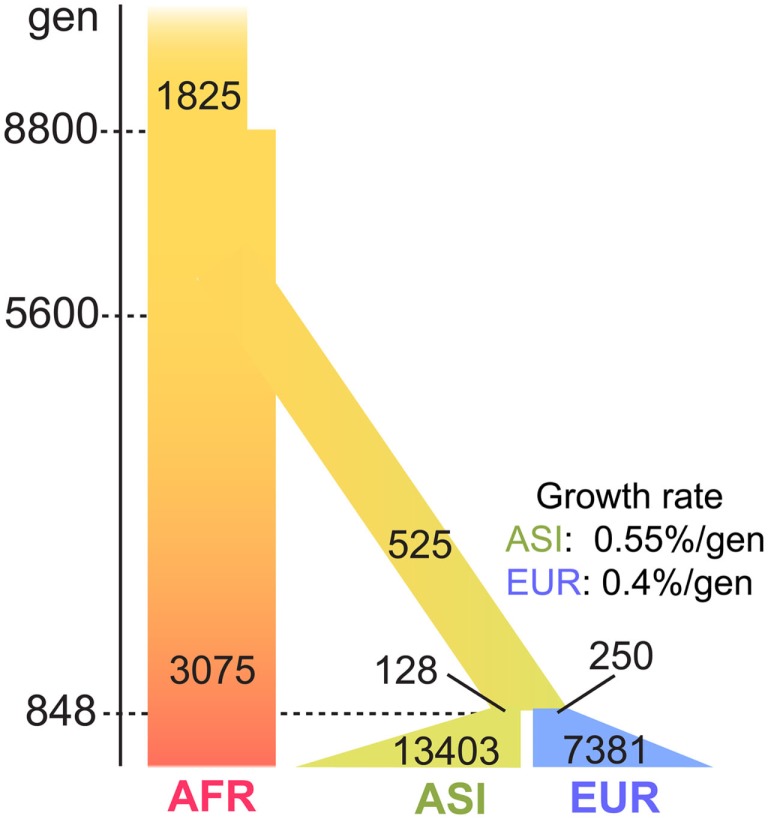
**The best-fit demographic model for the human mt genomes**. The parameters of the model are illustrated in the figure: passage of time is shown in the left side bars, with the most recent at the bottom. The population growth for ASI and EUR starts 848 generations ago from the present. The growth rate is also shown in the figure: the width of bars represents the size of the effective population. The population size was adjusted for mt genomes, under an assumption that the sex ratio is 1:1.

The short genome size and the high mutation rate of mtDNA could limit inference of the accurate demographic history. We therefore tested three demographic models (Marth et al., 2004; Voight et al., 2005; Gutenkunst et al., 2009) to determine the best-fit model for the 7,098 mt genomes (see Materials and Methods; Figure A1 in Appendix). From the comparison of the Π values of three populations of the 7,098 genomes and the simulated genomes, we determined Gutenkunst et al.’s (2009) model to be the best-fit model for our dataset (Table A3 in Appendix; Figure 4). This model includes the exponential population growth that followed severe population bottlenecks for the non-Africans population, which is consistent with the distribution of variants of our datasets.

### The estimation of the frequency of deleterious mutations

**Figure 5:**
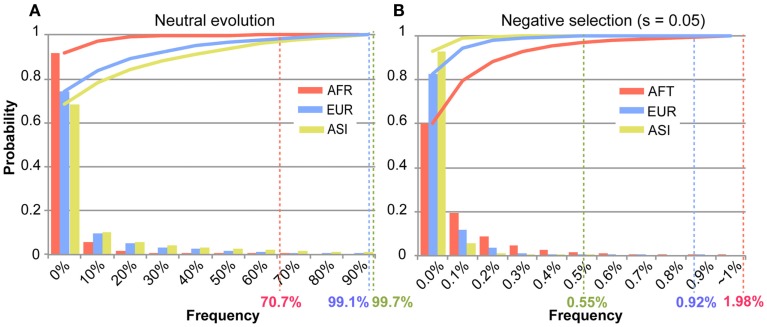
**The distribution of the frequency of a mutation among the simulations**. The *X*-axis represents bins of the frequency of a mutation in each population. The *Y*-axis indicates the occurrence of each bin of the mutation frequency in the simulated 10,000 datasets of mutation frequency. Each bar graphs the occurrence of the mutation frequency, and the solid line represents the accumulation of those occurrences for each population of AFR (red), ASI (yellow), and EUR (blue). The highest frequency data among 10,000 datasets was defined to “threshold frequency.” The threshold frequency in each population is shown below the *X*-axis, designated by the colored percentile figures. **(A)** Illustrates the threshold frequency under neutral evolution. **(B)** Illustrates the threshold frequency under negative selection (*s* = 0.05).

**Figure A3:**
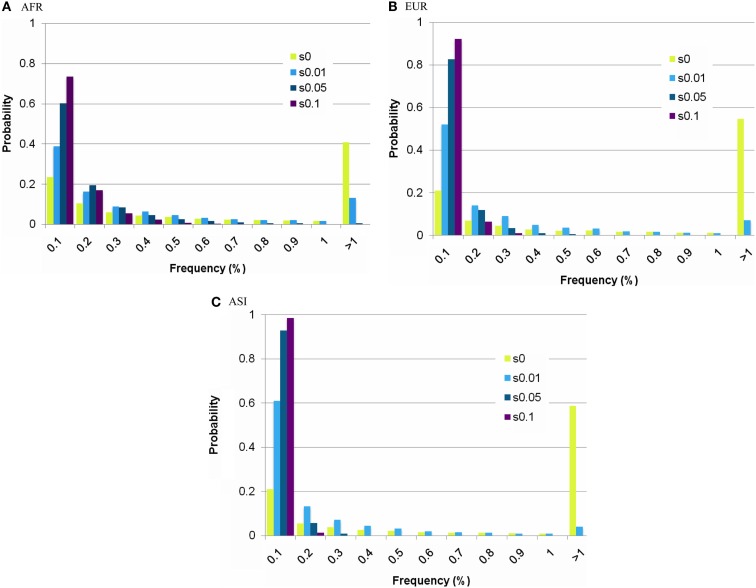
**Estimation of the frequency of a deleterious mutation**. **(A)** AFR, **(B)** EUR, **(C)** ASI. The *X*-axis indicates the frequency of a deleterious mutation, and the *Y*-axis indicates the probability of the frequency, from simulation studies. The probability changed depending on the selection coefficient (*s* = 0∼0.1).

For the identification of disease-related mutations among mt variants in the human population, we intended to ascertain the frequency of a deleterious mutation within the demographic history of the AFR, ASI, and EUR populations through the use of simulation studies. Based on the demographic model determined, we performed forward simulations for AFR, ASI, and EUR, separately, to ascertain the frequency of a deleterious mutation in each population (see details in Materials and Methods). These simulations generated 10,000 datasets regarding the frequency of mutation for each population. The occurrence of the frequency of the mutations among the datasets became our empirical probability which we used to ascertain the frequency of a deleterious mutation (Figure A3 in Appendix). In this simulation, the frequency increased from zero to one. The highest frequency among 10,000 datasets was defined as the “threshold frequency” of a mutation (Figure 5). The higher frequency than the threshold frequency is unlikely occurred.

The threshold frequency was determined according to the level of selective constraint, *s*. Under neutral evolution (*s* = 0), the threshold frequency in AFR, ASI, and EUR was 70.7, 99.7, and 99.1%, respectively (Figure 5A). The AFR threshold was low compared to the non-AFR threshold because AFR had not experienced a population bottleneck and had maintained its large effective ancestral population size. The chance for the fixation of a mutation in the larger population is smaller, and then the high frequency of a new mutation was unlikely even under neutrality. On the other hand, EUR and ASI had small effective ancestral population sizes, following severe population bottlenecks, but recently their population sizes have exponentially increased. This dynamic change in population size might give a high likelihood of increased frequency of a neutral mutation due to genetic drift.

Under the constraint of negative selection, a mutation is limited in its ability to increase its frequency in any population. The frequency of a mutation dramatically decreases in any selective constrains, compared to a neutral mutation. We tested various level of selective coefficients: the higher selective coefficient resulted in the lower threshold frequency (Figure A3; Table A4 in Appendix). Our simulations traced a frequency of a new mutation during evolution. If the mutation is slightly deleterious, the behavior of the mutations is nearly neutral (Ohta, 1992). Under the weak selective pressure (*s* = 0.01), distribution of the frequency of a mutation was similar to that of the frequency of a neutral mutation (Figure A3 in Appendix). Here we focused on disease-related mutations in the mtDNA, which contains mostly coding sequences. Therefore, we chose *s* = 0.05 which is deleterious enough to predominate against random drift in its effect on mutation behavior.

In a selective constraint (*s* = 0.05), the distribution of the frequency data was concentrated in the rare frequency. Most frequencies (at least 80% of the frequency data) were lower than 0.1% in any population. The threshold frequency was determined to be 1.98, 0.55, and 0.92%, in AFR, ASI, and EUR, respectively (Figure 5B; Table A4 in Appendix). The difference in threshold frequency among the three populations resulted from their different demographic history. AFR demonstrated as lightly higher threshold frequency than non-AFR, and EUR also demonstrated a slightly higher threshold frequency than ASI. The population growth rates could cause the differences in the threshold frequency. The current population size in the demographic model was 3,075, 13,403, and 7,381 in AFR, ASI, and EUR, respectively. AFR had no change in its size for 8,800 generations and was the smallest in size among the three populations. Although the size of ASI was smaller than that of EUR at population bottleneck, the higher population growth rate of ASI resulted in the larger final population size. Most mutations recorded in the simulations occurred very recently, and the frequency of the mutations in ASI was likely very low in the large population. Moreover, it is likely that the large population size in very recent history increased the efficacy of the operation of negative selection on the new deleterious mutation in non-AFR. The selective constraint could have more effect on the frequency of a mutation than genetic drift in the very recent population history for the non-AFR population.

### The identifications of pathogenic mutations in the 7,098 mt genomes

To detect pathogenic mutations, we applied the threshold frequency for our dataset and used the 506 diseases-associated mt mutations listed in the MITOMAP^2^. Those mutations have been reported to be associated with diseases, and based upon consistency of independent studies, were categorized as “Reported,” “Unclear,” or “Conflicting.” Fifty-two of the 506 mutations were categorized as “Confirmed,” indicating that at least two or more independent laboratories have published reports on their pathogenicity. We used these confirmed mutations as positive controls in detection of pathogenic diseases.

**Figure 6:**
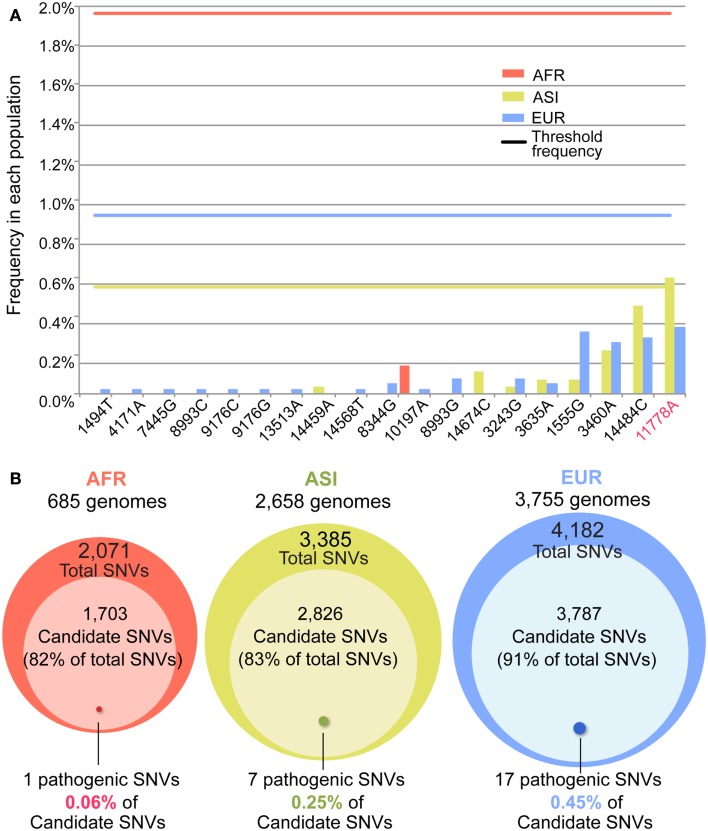
**The candidates and confirmed pathogenic mutations**. **(A)** The frequency of the 19 out of 52 confirmed pathogenic mutations. The *X*- and *Y*-axes indicate positions of each of 19 confirmed pathogenic mutations and its frequency in each population. A bar shows the frequency, and the line represents the threshold frequency. One mutation (11778A) showed the frequency higher than the threshold. **(B)** For each population, the candidate variants of a deleterious mutation in Dataset 1 were determined by using the threshold frequency. Among the candidates, the confirmed pathogenic mutations are indicated by the approximate size of Venn diagram.

**Figure A4:**
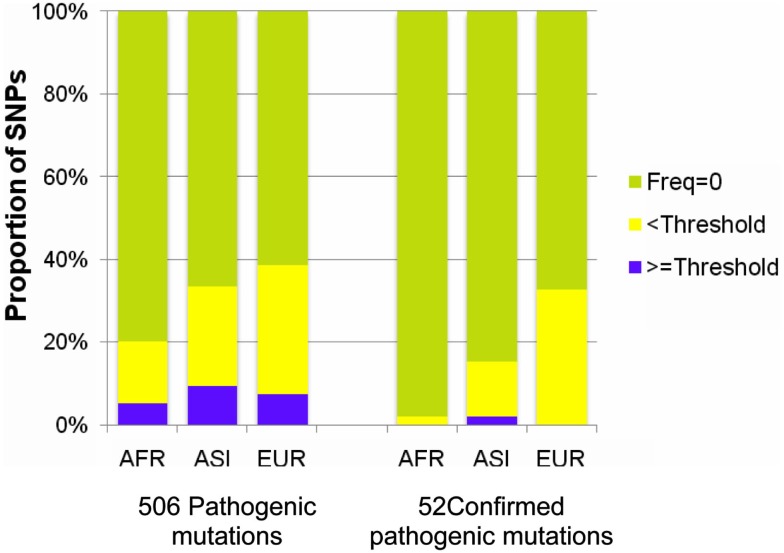
**Proportions of rare variants in the pathogenic mutations**. The 506 pathogenic and the 52 confirmed pathogenic mutations were categorized into three groups by frequency of a mutation in the 7,098 genomes: zero frequency (light green), lower than the threshold frequency (yellow), and larger than the threshold frequency (purple). The proportion of the 52 confirmed mutations with a frequency larger than the threshold was smaller than that of the 506 pathogenic mutations.

First, we examined the frequency of the 506 pathogenic mutations in our datasets. Among the mutations, 5, 9, and 7% showed a higher frequency than the threshold frequency in AFR, ASI, and EUR respectively (Figure A4 in Appendix). Of the 52 confirmed pathogenic mutations, 19 mutations were found in our datasets: 1, 7, and 17 mutations in AFR, ASI, and EUR respectively. Among the 19 mutations, 18 showed much lower frequency than threshold frequency, especially for AFR and EUR (Figure 6A). Only one mutation, 11778A, showing a higher frequency than the threshold in ASI, is one of the most well known pathogenic mutations, being the primary mutations of LHON (Wallace et al., 1988). In the case of LHON, factors in addition to the mutation may have an important role. In particular, the 11778A mutations showed an increase in the incidence of the disease penetrance along with the haplogroup J, which is of European origin (Brown et al., 1997; Carelli et al., 2006; Hudson et al., 2007; Ghelli et al., 2009). Therefore, selective constraint could fluctuate, depending upon the haplotypes. Interestingly, this effect of haplotype is consistent with our finding that the frequencies of the mutations are lower than the threshold in EUR but not in ASI. The mutations might be less deleterious in the ASI haplotypes and could increase in frequency in ASI.

Subsequently, we identified candidates for deleterious variants in the Datasets 1 and 2 SNVs. We assumed that the nucleotide sequence of *Homo neanderthalensis* (NC_011137) had an ancestral type and calculated frequency of derived type for each SNV in each population. For Dataset 1, the numbers of AFR, ASI, and EUR SNVs showing a frequency lower than the threshold were respectively 1,703 (82%), 2,826 (83%), and 3,787 (91%; Figure 6B; Table A5 in Appendix). For Dataset 2, 1,369 (77%), 2,112 (78%), and 2,735 (87%) SNVs in the three respective populations had a frequency below the threshold. Surprisingly, most of the SNVs in our dataset were candidates for deleterious variants. Among them, the proportion of confirmed pathogenic mutations that we detected in AFR, ASI, and EUR Dataset 1 SNVs, was very low, 0.06, 0.25, and 0.45% in the three respective populations (Figure 6B). We found a similarly small proportion in Dataset 2 (Table A5 in Appendix). Even considering all 506 pathogenic mutations, they represented only a small subset of candidates, about 1, 6, and 11% for AFR, ASI, and EUR, respectively. This low ratio of pathogenic mutations detected among the candidates was apparently an outcome of the large proportion of rare variants in the human population.

As mentioned before, rapid expansions of the human population have resulted in an excess of rare variants. The large number of these rare variants can be a major factor in limiting the possible detection of pathogenic mutations. In conclusion, our study showed that the recent population history of humans limits the detection of pathogenic mutations in mt genomes.

## Discussion

In this study, we analyzed 7,098 human mt genomes and identified the largest number of SNVs in the mt genomes (Figure 1). Most of the identified variants were rare, and a much smaller proportion of rare compared to common variants was shared across the AFR and non-AFR populations (Figures 2 and 3). The main factor in this distribution of variants was the incidence of recent population bottlenecks followed by exponential population growth. Under the assumption of population history (Figure 4), we estimated the threshold frequency of a deleterious mutation in the human population (Figure 5). Although the threshold frequency is very low, we detected a large number of candidate variants that potentially relate to diseases and examined the small proportion of the known pathogenic mutations within the candidates (Figure 6).

**Figure A5:**
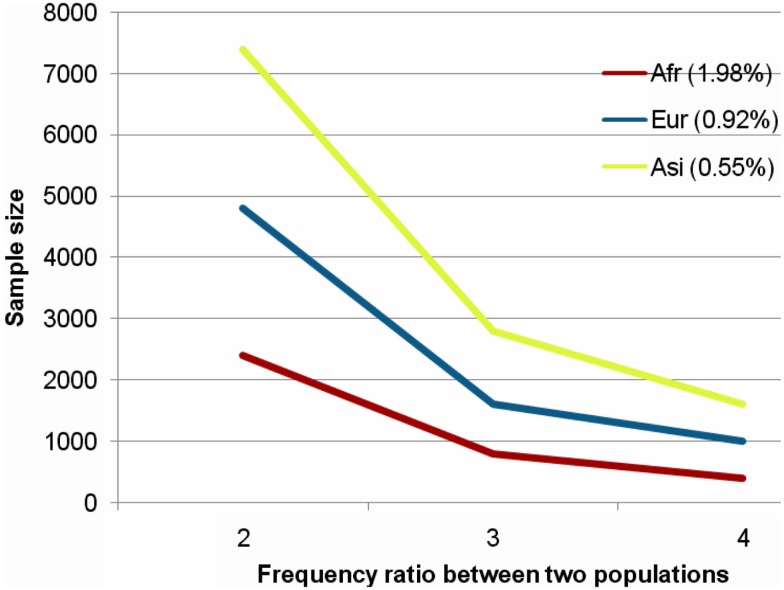
**Sample sizes for statistical power**. Supposing two populations of case and control, chi-square tests were carried out to test differences of the frequency of a mutation between two populations. Two populations have the same size. We fixed the frequency of 1.98, 0.92, and 0.55% (the threshold frequencies of AFR, EUR, and ASI) for the control population and supposed a ratio of frequency of case to control as 2, 3, and 4, indicated at *X*-axis. In each case, we gave a range of population sizes for the chi-square test, and the *Y*-axis represented total population size of case and control to show a significant difference (*P* < 0.01) of the frequency between two populations.

Only 19 pathogenic mutations in the 7,098 mt genomes were detected. The largest number of genomes carrying pathogenic mutations among those 19 mutations was 1/685, 12/2,685, and 13/3,755 in AFR, ASI, and EUR, respectively. To detect one genome with a pathogenic mutation, at least 685, 222, and 289 genomes of the three respective populations are needed. The sample size of 7,098 genomes was not sufficient for identifying 33 out of the 52 known pathogenic mutations. Detection of significant associations of rare variants with particular diseases in genome-wide association studies has been considered challenging (Asimit and Zeggini, 2010; Bansal et al., 2010). For example, if the ratio of the frequency of a candidate mutation of the case versus control populations is two, and the frequency of the mutation is the same as the threshold frequency, to attain the significant difference of the frequency of the mutation between the case population and the control population (*P* < 0.01, Fisher’s-exact test), total sample sizes of at least 2400, 4800, and 7400 are required for AFR, ASI, and EUR respectively (Figure A5 in Appendix). This suggests that a huge sample size is needed in order to detect a single pathogenic mutation.

**Figure A6:**
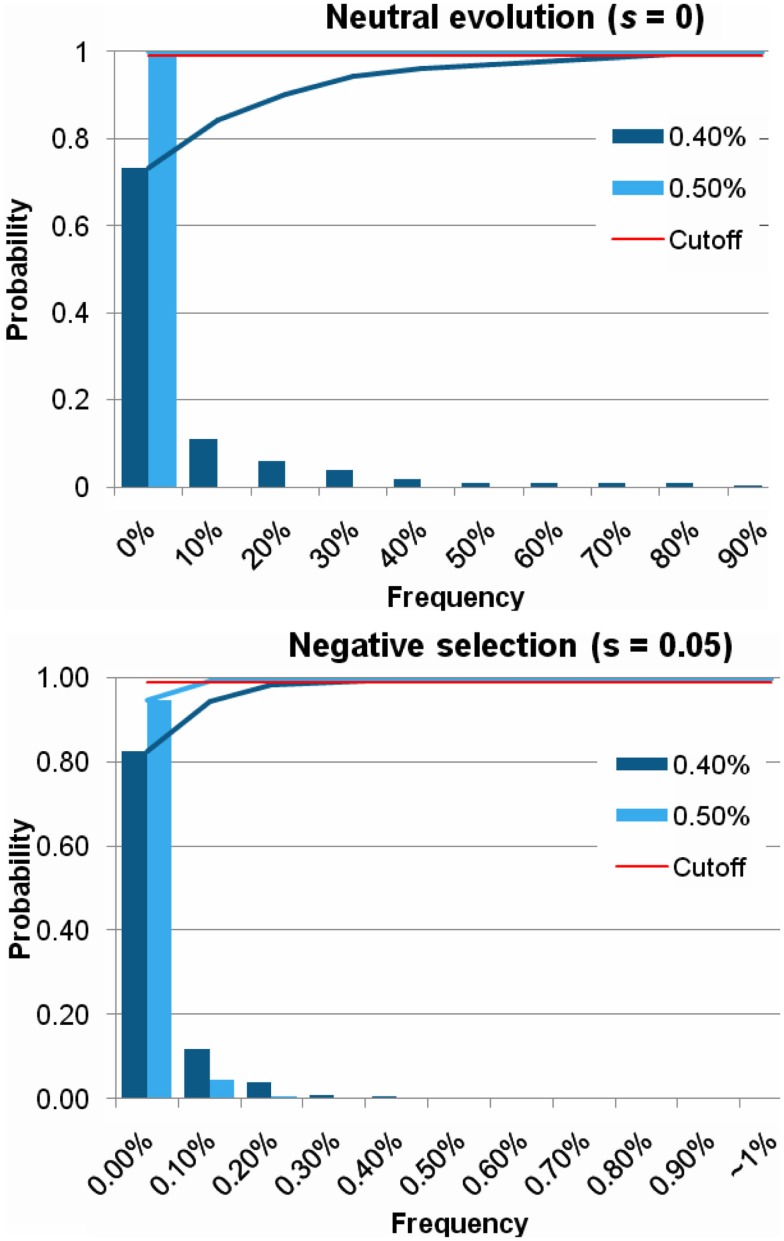
**Distribution of frequency of a mutation in various population growth rate**. For EUR, the parameter of population growth rate was 0.4% per generation in Gutenkunst’s model. We tested the larger growth rate, 0.5%, also. In both case of neutral evolution and negative selection, a frequency of a mutation decreased in the larger growth rate. The threshold frequency (*P* < 0.01) under negative selection (*s* = 0.05) was 0.19%, which was much lower than the threshold frequency in the simulation with 0.4% population growth rate (0.35%).

As noted throughout the manuscript, the main reason for pathogenic mutations being so rarely found in the human population is exponential population growth. Recent studies inferring the population history of humans have estimated various rates of population growth (Gutenkunst et al., 2009; Coventry et al., 2010; Gravel et al., 2011; Li and Durbin, 2011). The growth rate we used for EUR (Gutenkunst et al., 2009) was lower than the rate inferred by Coventry et al. (2010). We tested the higher growth rate and found that the chance of survival of a deleterious mutation was smaller. When we used the higher growth rate, the threshold frequency became lower (Figure A6 in Appendix). This suggests that our estimation of the threshold frequency is conservative and that the threshold to determine pathogenic mutations is likely to be much lower than our estimation for EUR.

In addition, our findings suggest that rare variants in the mt genomes could play a major role in causing mt diseases. Although most new mutations could be eliminated by genetic drift in an equilibrium population, such a population could also attain many new mutations due to increase in population size (Coventry et al., 2010). If population expansion was recent, these could include deleterious mutations, as insufficient time for elimination of such mutations by purifying selection would have passed (Lohmueller et al., 2008). We determined more than 83 and 91% of the variants that existed in the ASI and EUR population were rare enough to be candidates for deleterious variants. The proportions were larger than the proportion (82%) of candidates within the AFR, which has not experienced recent exponential population growth (Figure 6). The non-AFR population histories have resulted in a greater genetic load of rare variants within the populations. It has been suggested that the accumulation of rare variants in a genome could play a role in causes of complex diseases (Keinan and Clark, 2012). The accumulation of rare variants in the mt genomes also could be a contributing cause of common diseases.

Our study clearly showed the impact of population history on the detection of disease mutations in mt genomes and the difficulty of that detection. Our approach could produce some expectation regarding the detection of disease-related mutations in nuclear genomes. The previous study analyzed the nuclear variants in the data from the 1000 Genomes project (1000 Genomes Project Consortium et al., 2010) and showed a distribution of variants similar to that of our dataset: a large proportion of rare variants and a low proportion of shared variants across populations due to population growth (Gravel et al., 2011). The disease-related mutations in nuclear genomes have been believed to be rarely found in the human population (Manolio et al., 2009; Cirulli and Goldstein, 2010). The rarity of the mutations responsible for diseases can give rise to difficulties in detecting the mutation in nuclear genomes too. To apply our approach to nuclear genomes, it is necessary to incorporate other representative factors, such as recombination. Recombination does not occur randomly across genomes, causing various selection constraints. The low nucleotide diversity of the nuclear genome compared to that of the mt genome means that an extraordinarily large sample size is required to detect rare variants. With these factors in mind, future studies can apply our approach to nuclear variants for the discovery of pathogenic mutations.

## Conflict of Interest Statement

Our research was conducted in the absence of any commercial or financial relationships that could be construed as a potential conflict of interest.

## Supplementary Material

The Supplementary Material for this article can be found online at http://www.frontiersin.org/Evolutionary_and_Population_Genetics/10.3389/fgene.2013.00013/abstract

## Footnotes

^1^www.mtdb.igp.uu.se

^2^www.mitomap.org

## Appendix

**Table A1:** **The frequency of mitochondrial haplogroups**.

Haplogroup	No. of haplotypes	No. of genomes	Freq.
L0	43	121	0.017
L1	33	107	0.015
L2	45	172	0.024
L3	74	241	0.034
L4	8	23	0.003
L5	7	14	0.002
L6	2	7	0.001
AFR (L)	212	685	0.096

M	309	1250	0.176
C	70	318	0.045
D	173	781	0.110
E	13	86	0.012
G	28	136	0.019
Q	9	29	0.004
Z	15	58	0.008
ASI (M)	668	2658	0.374

N	47	177	0.025
A	49	286	0.040
F	30	109	0.015
B	85	290	0.041
H	139	816	0.115
HV	15	104	0.015
I	12	44	0.006
J	50	213	0.030
K	60	279	0.039
O	2	4	0.001
P	17	33	0.005
R	93	279	0.039
S	5	11	0.002
T	35	173	0.024
U	166	630	0.089
V	19	103	0.015
W	20	88	0.012
X	33	89	0.013
Y	6	27	0.004
EUR (N)	832	3755	0.529

Non-African (M&N)	1500	6413	0.034

Total	1712	7098	1.000

**Table A2:** **The frequency and nucleotide diversity (Π) of each population**.

Population	No. of genomes	Dataset 1		Dataset 2	
		Π	Max	Π	Max
All	7,098	39.8	123	39.3	122
AFR^a^	685 (10%)	64.0	123	63.3	122
Non-AFR^b^	6,413 (90%)	35.4	71	34.9	68
ASI^c^	2,658 (37%)	30.1	62	29.6	61
EUR^d^	3,755 (53%)	30.6	66	30.1	62
Bet. AFR and ASI		57.9	118	57.3	116
Bet. AFR and EUR		60.3	122	59.7	117
Bet. ASI and EUR		40.6	71	40.2	68
Bet. AFR and non-AFR		59.3	122	58.7	117

*^a^L haplogroups*.

*^b^M and N haplogroups*.

*^c^M haplogroups*.

*^d^N haplogroups*.

**Table A3:** **Comparisons of three demographic models**.

	All	AFR	Non-AFR	ASI	EUR
7,098 genomes	7,098	685	6,413	2,658	3,755
Observation	39.3	63.3	34.9	29.6	30.1

Gutenkunst et al.					
**Mean**	**50.6**	**66.5**	**28.9**	**11.4**	**32.4**
SD	11.8	28.7	6.3	3.8	7.9
Difference	10.1	1.2	−6.7	−22.4	4.8

Voight et al.					
**Mean**	**69.0**	**62.2**	**62.9**	**54.6**	**54.7**
SD	27.6	29.6	27.1	24.3	24.3
Difference	29.6	−1.1	27.9	24.9	24.5

Marth et al.					
**Mean**	**90.4**	**63.1**	**86.0**	**63.9**	**65.5**
SD	29.9	29.6	29.7	31.5	24.0
Difference	51.1	−0.2	51.1	34.2	35.4

*Thousand sets of mt genome sequences were simulated based on each the three models, and then the mean of the Π values of the 1,000 sets of simulated genomes were compared with the corresponding Π values of the 7,098 genomes. The difference corresponds to the extent of subtraction of the simulation from the observation*.

*Gutenkunst et al.’s model showed the closest Π values to the observation. The differences were within the standard deviation of 1,000 sets of Π of the simulated genomes, with the exception of the ASI’s mean Π. The mean Π of ASI of the simulation was much smaller than that of the observation. The parameters for ASI in this model were inferred from the CHB (Han Chinese) samples, whereas ASI included the genomes originated from more diverse Asian populations. Thus it is likely that the mean Π for ASI in the simulation is smaller than the observed Π. The other models of Marth et al. (2004) and Voight et al. (2005) resulted in too-large Π values compared to the observation, especially for non-AFR*.

**Table A4:** **Estimation of the threshold frequency of a deleterious mutation in each population**.

Population	Effective population size	Neutral	0.01^a^	0.05^a^	0.1^a^
			Frequency (*P* < 0.01)		
AFR	3,075	70.7%	8.9%	1.98%	1.07%
ASI	13,403	99.7%	15.9%	0.55%	0.26%
EUR	7,381	99.1%	18.9%	0.92%	0.49%

	**Sample size**		**The number of genomes**		

AFR	685	484	61	14	7
ASI	2,658	2,651	423	15	7
EUR	3,755	3,720	710	35	18

*^a^Selection coefficient*.

*We estimated the frequency of a deleterious mutation in each population by simulations as shown in the upper part of the table. The number of genomes in the sample size which was the same size of our data set (685 AFR, 2,658 ASI, and 3,755 EUR genomes) was calculated from the threshold frequency, and was shown in the bottom part of the table*.

**Table A5:** **The proportion of the rare SNVs**.

	Population	AFR	ASI	EUR
	Threshold frequency (%)	1.98	0.55	0.92
	No. of genomes	685	2,568	3,755
Dataset 1	No. of total SNVs	2,071	3,385	4,182
	No. of candidate SNVs	1,703	2,826	3,787
	Proportion of candidates in total SNVs (%)	82	83	91
	No. of pathogenic mutations	75	121	159
	No. of confirmed pathogenic mutations	1	7	17
	Proportion of confirmed pathogenic mutations in candidates (%)	0.06	0.25	0.45
Dataset 2	No. of total SNVs	1,778	2,701	3,141
	No. of candidate SNVs	1,369	2,112	2,735
	Proportion of candidates in total SNVs (%)	77	78	87
	No. of pathogenic mutations	72	108	133
	No. of confirmed pathogenic mutations	1	6	9
	Proportion of confirmed pathogenic mutations in candidates (%)	0.07	0.28	0.33

*The number of rare SNVs in the 7,098 genomes was the number of SNVs showing a frequency lower than the threshold frequency (Candidate SNVs). Red characters represent the proportion of the rare SNVs among total SNVs in the datasets*.
